# A Complex Set of Sex Pheromones Identified in the Cuttlefish *Sepia officinalis*

**DOI:** 10.1371/journal.pone.0046531

**Published:** 2012-10-30

**Authors:** Jérémy Enault, Céline Zatylny-Gaudin, Benoît Bernay, Benjamin Lefranc, Jérôme Leprince, Michèle Baudy-Floc'h, Joël Henry

**Affiliations:** 1 FRE CNRS 3484 BIOMEA, Biologie des Mollusques Marins et des Ecosystèmes Associés, Université de Caen Basse-Normandie, Caen, France; 2 Post Genomic platform PROTEOGEN, Université de Caen Basse-Normandie, IFR ICORE 146, Caen, France; 3 INSERM U982, Différenciation et Communication Neuronale et Neuroendocrine, PRIMACEN, IFRMP23, Université de Rouen, Mont-Saint-Aignan, France; 4 UMR CNRS 6226, Ciblage et Auto-Assemblages Fonctionnels, Sciences Chimiques de Rennes, Université de Rennes I, Av. du Général Leclerc, Rennes, France; Macquarie University, Australia

## Abstract

**Background:**

The cephalopod mollusk *Sepia officinalis* can be considered as a relevant model for studying reproduction strategies associated to seasonal migrations. Using transcriptomic and peptidomic approaches, we aim to identify peptide sex pheromones that are thought to induce the aggregation of mature cuttlefish in their egg-laying areas.

**Results:**

To facilitate the identification of sex pheromones, 576 5′-expressed sequence tags (ESTs) were sequenced from a single cDNA library generated from accessory sex glands of female cuttlefish. Our analysis yielded 223 unique sequences composed of 186 singletons and 37 contigs. Three major redundant ESTs called SPα, SPα′ and SPβ were identified as good candidates for putative sex pheromone transcripts and are part of the 87 unique sequences classified as unknown. The alignment of translated SPα and SPα′ revealed a high level of conservation, with 98.4% identity. Translation led to a 248-amino acid precursor containing six peptides with multiple putative disulfide bonds. The alignment of SPα-α′ with SPβ revealed a partial structural conservation, with 37.3% identity. Translation of SPβ led to a 252-amino acid precursor containing five peptides. The occurrence of a signal peptide on SPα, SPα′ and SPβ showed that the peptides were secreted. RT-PCR and mass spectrometry analyses revealed a co-localization of transcripts and expression products in the oviduct gland. Preliminary *in vitro* experiments performed on gills and penises revealed target organs involved in mating and ventilation.

**Conclusions:**

The analysis of the accessory sex gland transcriptome of Sepia officinalis led to the identification of peptidic sex pheromones. Although preliminary functional tests suggested the involvement of the α3 and β2 peptides in ventilation and mating stimulation, further functional investigations will make it possible to identify the complete set of biological activities expected from waterborne pheromones.

## Background

The French cuttlefish *Sepia officinalis* is a nectobenthic cephalopod that performs horizontal migrations [1]. During reproduction, which takes place after a two-month long migration from winter areas, cuttlefish aggregate in specific mating and spawning coastal areas.

In most molluscs, chemical cues are important for social communication [2]. Chemical communication in cuttlefish was demonstrated by Boal and collaborators [3], [4].using y-mazes.

The occurrence of chemical messengers released by the genital apparatus and by the egg mass has long been thought to explain the aggregation observed between April and June [4]. Ovarian regulatory peptides that stimulate oviduct and accessory sex gland contractions have already been identified in *Sepia officinalis* [5] [6] [7] [8] [9].

We demonstrated that *Sepia* oocytes were responsible for the expression and secretion of waterborne regulatory peptides released in the genital tract during oocyte transport and in the mantle cavity during egg capsule secretion. These peptides modulate the contractions of the oviduct during ovulation and. when they reach the mantle cavity (along with the oocytes), they induce the release of secretions corresponding to egg capsule) targeting the oviduct gland and main nidamental glands. The low molecular mass of these peptides (500–1,500 Da), and the absence of disulfide bonds combined with the absence of N and C-terminal protections, result in low structural stability and a short half-life in natural environments.

But messengers implied in behavioral modifications and in the stimulation of mating and of gamete release could be large peptides or polypeptides, as already described in the marine gastropod *Aplysia* [10] [11] [12].

Indeed, field studies [13] [14] [15] [16] [17] have shown that *Aplysia* are solitary animals most of the year, but move into breeding aggregations during the reproductive season when they mate and lay eggs. After ovulation, the albumen gland wraps eggs into a long, string-like cordon that has a high surface-to-volume ratio. These egg cordons are a source of both waterborne and contact pheromones that attract mates and induce mating and egg-laying [11] [17] [18].

A waterborne pheromonal attractant (attractin) was first isolated from eluates of *Aplysia californica* egg cordons and characterized [11]. It is a 58-residue peptide with a single N-glycosylation and three intra-chain disulfide bonds [17] [19] [20]. Although the precursor contains a single copy of attractin [21], its transcripts and its expression products are very abundant in the albumen gland where they reach 20% of the transcripts [22].

Moreover, T-maze assays showed that attractin acted as part of a bouquet of waterborne odors [11] [21] composed of enticin (7.7 kDa) [22], temptin (10.9 kDa) [22] and seductin (28.9 kDa) [23].

In *Sepia officinalis*, mating is a stereotyped behavior during which spermatophores are deposited by males into a copulatory pouch that is located below the females' beak and stores sperm prior to fertilization. The oviduct gland and the main nidamental glands, which play the same role as the albumen gland in *Aplysia*, secrete two layers of capsule that embed oocytes during egg-laying just before fertilization.

In the present study, our investigations focused on the identification and the characterization of peptides and polypeptides involved both in mature adult aggregations on coastal egg-laying areas and in mating stimulation. A cDNA library was created from oviduct glands and main nidamental glands and yielded 576 expressed sequence tags (ESTs) that allowed us to identify the main expression products secreted during egg-laying on the basis of clone redundancy and signal peptide occurrence.

## Results

### Reproduction stage-specific cDNA library, EST assembly and Assignment of putative gene functions using Gene Ontology

A directional cDNA library was created from the accessory sex glands of three adult females.

A total of 576 randomly selected clones were single-pass sequenced from the 5′ end, then pre-processed to remove poor-quality sequences and cloning-vector sequences. Finally, 560 high-quality ESTs ranging from 350 to 1,280 bp, with an average length of 975 bp, were obtained.

Redundant ESTs were assembled into overlapping contigs using CAP3 software, which yielded 223 unique sequences: 37 contigs and 186 singletons with a redundancy of about 66.7%. The results of the *in silico* analysis are summarized in tables 1 and 2.

**Table 1:** General characteristics of *Sepia officinalis* accessory sex gland ESTs.

Total number of **sequenced** cDNA	**576**
Number of high quality ESTs^a^	**560**
Average length of high quality ESTs (bp)	**974.6**
Number of contigs^b^	**37**
Number of ESTs in contigs	**374**
Number of singletons^c^	**186**
Redundancy^d^	**66.7%**

a length of sequences used for comparison after editing (<100-bp et N-rich inserts were excluded).

b ESTs with minimum 90% identity over 100-bp region were clustered together, forming a cluster.

c that did not sufficiently match any sequence in the data set to allow assembly.

d number of ESTs assembled in clusters/total ESTs.

**Table 2:** BLASTX searches and analyses for the collection of *Sepia officinalis* contigs and singletons.

Number of unique sequences	**223**
Number of unique sequences with BLASTX hits	**129** (57.8%)
Number of unique sequences with predicted signal peptides	**56** (25.1%)
Number of contigs containing :	
2 ESTs	**23**
3 ESTs	**5**
4 ESTs	**3**
5 ESTs	**1**
>6 ESTs	**5**

The 223 EST unigene sequences were compared by BLASTX to various databases using the substitution matrix BLOSUM62 [24]. Using a cut-off expect-value of E<10^−5^, 136 sequences (61%) were found to match with reported sequences and 87 (39%) were classified as unknown. 11 sequences (5%) were highly similar to cephalopod genes identified from the genera *Sepia, Loligo, Octopus, Ommastrephes* and *Enteroctopus*.

As the main expression products of accessory sex glands are extracellular secreted proteins, the occurrence of signal peptides was checked and yielded 56 (25%) unique secreted sequences.

Sixty-two percent of the contigs were assembled from two ESTs and 13% from more than five ESTs. Two contigs were assembled from over 10 ESTs: one coding for a glutathione S-transferase and one for procollagen type XII α-1.

**Figure 1:**
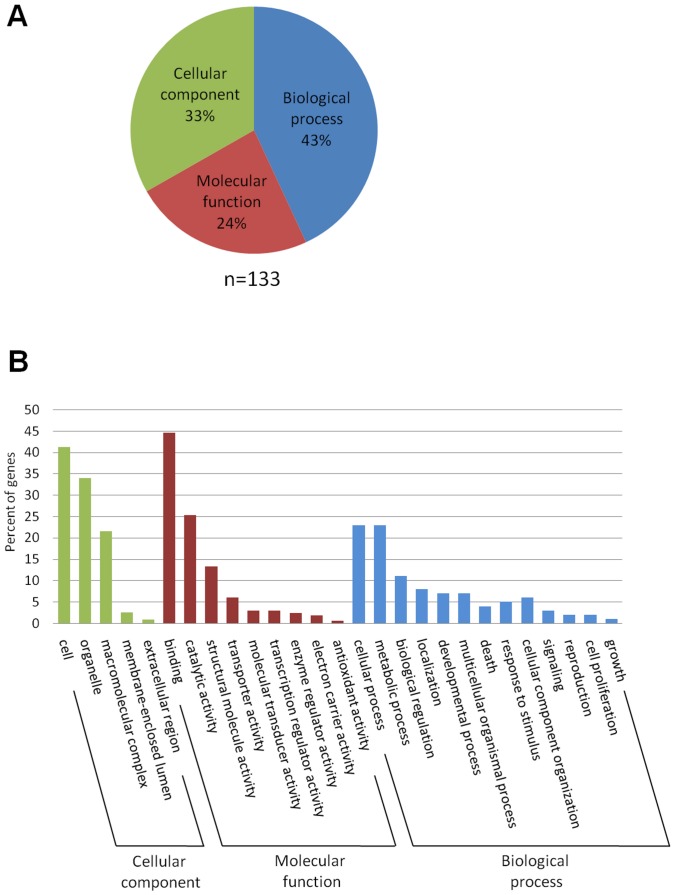
Functional categories by Gene Ontology analysis of the sex accessory gland sequences. (A) 133 sequences out of a total of 560 sequences; not all unigenes could be annotated and some received multiple annotations. (B) Comparison of different percentages in each GO sub-category is represented in all categories.

Among the 136 identified sequences, 133 were successfully processed by Blast2GO and then associated to putative functions (figure 1).

GO annotations fall into three independent categories : Biological process, Molecular function, and Cellular component. As shown in figure 1A, 43% of annotated ESTs were associated to «biological process» versus 33% to «cellular components» and 24% to «molecular functions».

In the “molecular functions” category (figure 1B), ESTs displaying a binding function (45%) predominated, followed by those displaying a catalytic activity (25%). In the “biological process” category (figure 1C), ESTs involved in cellular process and metabolic process were predominant (46%).

The “reproduction” group contributed to a total of approximately 1% of the total number of identified GO terms.

In the “cellular component” category (figure 1D), ESTs functionally involved in the cell predominated (41%), followed by those functionally displayed in organelles (34%). It should be noted that most of this category consists of the diverse sub-units of ribosomal proteins which are represented by 20 unique sequences.

### Identification of sex pheromone ESTs

The identification of good candidates for sex pheromone transcripts was based on three main criteria corresponding to sex pheromones described in *Aplysia* [19] [22]: transcript redundancy, occurrence of a signal peptide and structure of the putative expression products. Out of 560 high quality ESTs, 168 were identified by Blast and 392 were considered as unknown. 68% of the unknown ESTs (268) corresponded to 3 major redundant ESTs leading to three proteic precursors called SPα (66 ESTs, 11.8%), SPα′ (65 ESTs, 11.6%) and SPβ (134 ESTs, 23.9%) (SP is for sex pheromones).

**Figure 2:**
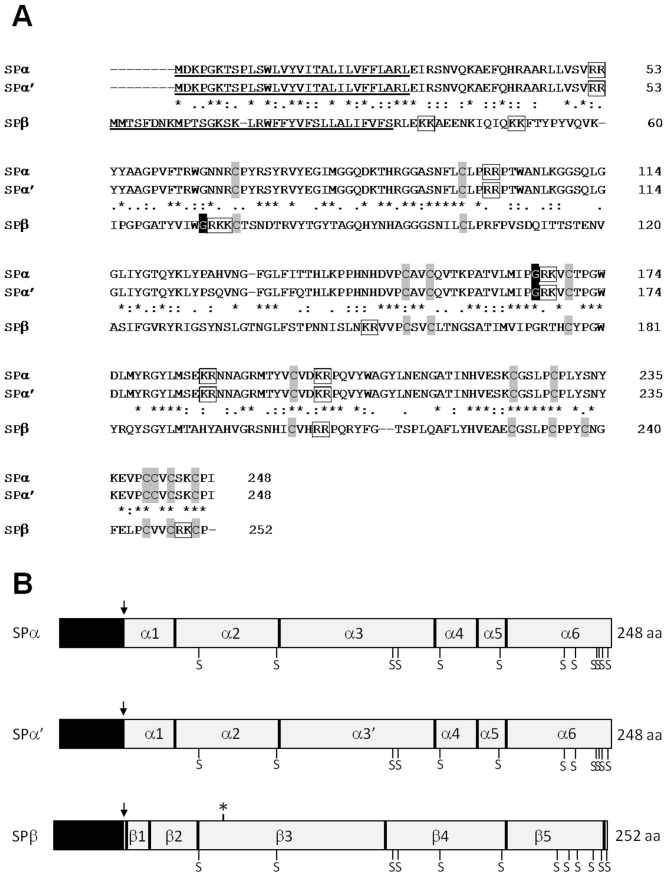
Description of sex pheromone precursors. (A) Amino acid alignments of the 3 precursors. Asterisks (*) indicate strict identity between sequences; colons (:) indicate strong similarity; dots (.) indicate weak similarity; and hyphens (-) represent alignment gaps. Predicted signal sequences are underlined. Cysteines are filled in grey; putative amidations are filled in black; and potential basic residue cleavage sites are boxed. (B) Schematic diagrams showing the organization of *Sepia officinalis* pheromone precursors. Precursors encode a complex cocktail of peptides and polypeptides resulting from dibasic cleavages. Black box, signal peptide; arrow, predicted site of signal sequence cleavage; vertical black line, potential dibasic residue cleavage site; asterisk, predicted N-linked glycosylation site; S, Cys residue.

While SPα and SPα′ shared a high level of homology (98.4% identity and 99.6% similarity), SPβ appeared to be partially conserved with only 37.3% identity and 56.7% similarity with SPα/SPα′. SPα, SPα′ and SPβ are three related precursors (figure 2) that represent 47.8% of the sequenced ESTs and 68.4% of the unknown category.

SPα and SPα′ encode a protein precursor of 248 amino acids with an N-terminal signal peptide of 29 residues. The dibasic processing of the precursors led to six putative peptides ranging from 1.3 to 7 kDa, summarized in table 3. The 7 kDa peptide is C-terminally amidated and two cysteines are thought to form an intrachain disulfide bond. The occurrence of interchain disulfide bonds cannot be ruled out. The sequence divergence observed between SPα and SPα′ is located in the 7 kDa peptide (64 amino acids) with two couples of substituted amino acids.

**Table 3:** Peptides resulting from dibasic cleavages of SP precursors.

SP Precursor	Primary sequences of putative expression products processed from dibasic cleavages	Putative post-translational modifications	Monoisotopic m/z
**SPα SPα′**			
peptide α1	EIRSNVQKAEFQHRAARLLVSV	/	2551.42
peptide α2	YYAAGPVFTRWGNNRCPYRSYRVYEGIMGGQDKTHRGGASNFLCLP	disulfide bond	5226.49
peptide α3	PTWANLKGGSQLGGLIYGTQYKLYPAHVNGFGLFITTHLKPPHNHDVPCAVCQVTKPATVLMIPa	C-terminal amidation and disulfide bond	6906.61
peptide α3′	PTWANLKGGSQLGGLIYGTQYKLYPSQVNGFGLFFQTHLKPPHNHDVPCAVCQVTKPATVLMIPa	C-terminal amidation and disulfide bond	6974.60
peptide α4	VCTPGWDLMYRGYLMSE	disulfide bond	2020.89
peptide α5	NNAGRMTYVCVD	disulfide bond	1342.59
peptide α6	PQVYWAGYLNENGATINHVESKCGSLPCPLYSNYKEVPCCVCSKCPI	disulfide bond	5171.37
**SPβ**			
peptide β1	AEENKIQIQ	/	1072.56
peptide β2	FTYPYVQVKIPGPGATYVIWa	C-terminal amidation	2298.23
peptide β3	CTSNDTRVYTGYTAGQHYNHAGGGSNILCLPRFPVSDQITTSTENVASIFGVRYRIGSYNSLGTNGLFSTPNNISLN	N-glycosylation and disulfide bond	8256.97
peptide β4	VVPCSVCLTNGSATIMVIPGRTHCYPGWYRQYSGYLMTAHYAHVGRSNHICVH	disulfide bond	5901.80
peptide β5	PQRYFGTSPLQAFLYHVEAECGSLPCPPYCNGFELPCVVCRKCP	disulfide bond	4913.27

The 134 cDNA clones of SPβ contained an open reading frame that encoded a 252-amino acid protein precursor with a 34-residue signal sequence. The dibasic processing of the precursor yielded seven putative peptides ranging from 1.1 to 8.3 kDa, summarized in table 3. Among these putative peptides, only a 2.3-kDa one appeared to be C-terminally amidated.

### Tissue mapping

**Figure 3:**
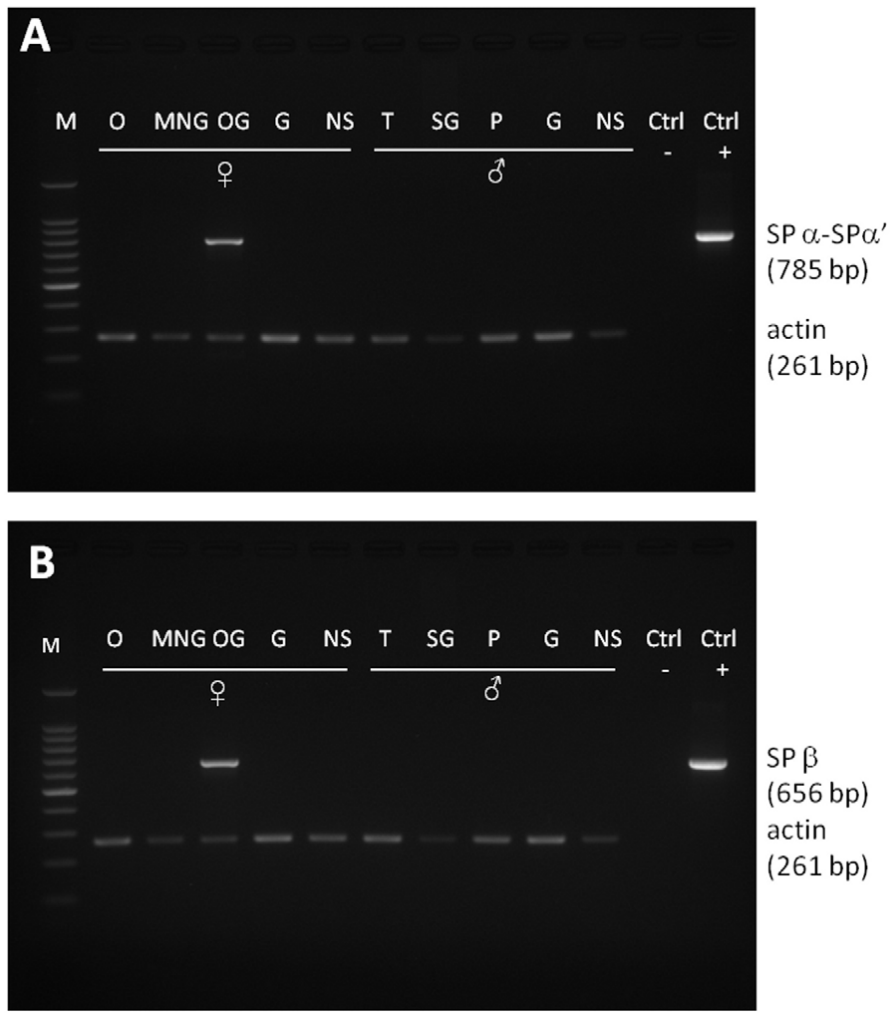
Tissue-specific patterns of expression for candidate pheromones assayed via reverse transcription PCR. Expression patterns for SPα, SPα′ and SPβ transcripts and actin were assayed from three females and three males. Gels (A) and (B) correspond to SPα-α′ and SPβ, respectively. M, weight marker; O, ovary; MNG, main nidamental gland; OG, oviduct gland; G, gills; NS, nervous system; T, testis; SG, secondary glands; P, Penis; Ctrl−, negative control (no template added to PCR mix); Ctrl+, positive control (the template was a plasmid containing the insert of interest).

RT-PCR performed from male and female tissues revealed localization of SP transcripts restricted to female accessory sex glands. SPα, SPα′ and SPβ were detected in the oviduct gland (figure 3).

**Figure 4:**
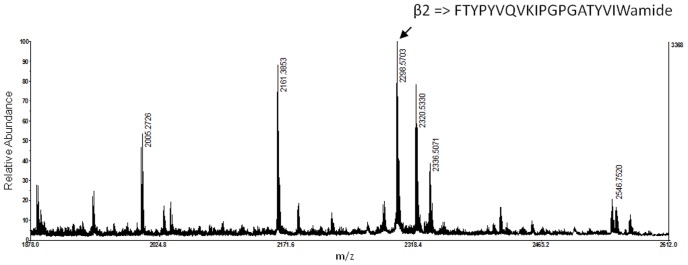
MALDI-TOF MS spectrum of β2 peptide recovered from the oviduct gland.

A preliminary run of MALDI-TOF-MS analysis yielded several expected expression products (β1, β2 and β4 peptides) demonstrating a dibasic proteolytic processing of the precursors. The MS spectrum of the β2 peptide is shown in figure 4. In addition, the identity of the β2 peptide (m/z 2298.2) was confirmed by MS/MS and by screening the translated EST library of accessory sex glands.

Several m/z results corresponding to internal cleavages of the α3 and α3′ peptides demonstrated that the classical dibasic processing cleavages were followed by monobasic cleavages and by unusual cleavages leading to a set of N-terminally truncated peptides (table 4).

**Table 4:** MALDI-TOF detected ions resulting from truncated α3 and α3′ peptides.

Designation	Primary sequences	Detected ion	Average m/z
truncated α3	WANLKGGSQLGGLIYGTQYKLYPAHVNGFGLFITTHLKPPHNHDVPCAVCQVTKPATVLMIPa	4M^3+^	8977.8
truncated α3	NLKGGSQLGGLIYGTQYKLYPAHVNGFGLFITTHLKPPHNHDVPCAVCQVTKPATVLMIPa	4M^3+^	8604.6
truncated α3	GGSQLGGLIYGTQYKLYPAHVNGFGLFITTHLKPPHNHDVPCAVCQVTKPATVLMIPa	M^+^	6100.4
truncated α3	LYPAHVNGFGLFITTHLKPPHNHDVPCAVCQVTKPATVLMIPa	4M^3+^	6101.7
truncated α3′	WANLKGGSQLGGLIYGTQYKLYPSQVNGFGLFFQTHLKPPHNHDVPCAVCQVTKPATVLMIPa	4M^3+^	9036.1
truncated α3′	LKGGSQLGGLIYGTQYKLYPSQVNGFGLFFQTHLKPPHNHDVPCAVCQVTKPATVLMIPa	M^+^+Na^+^	6429.5

### Bioactivity of C-terminally amidated peptides

**Figure 5:**
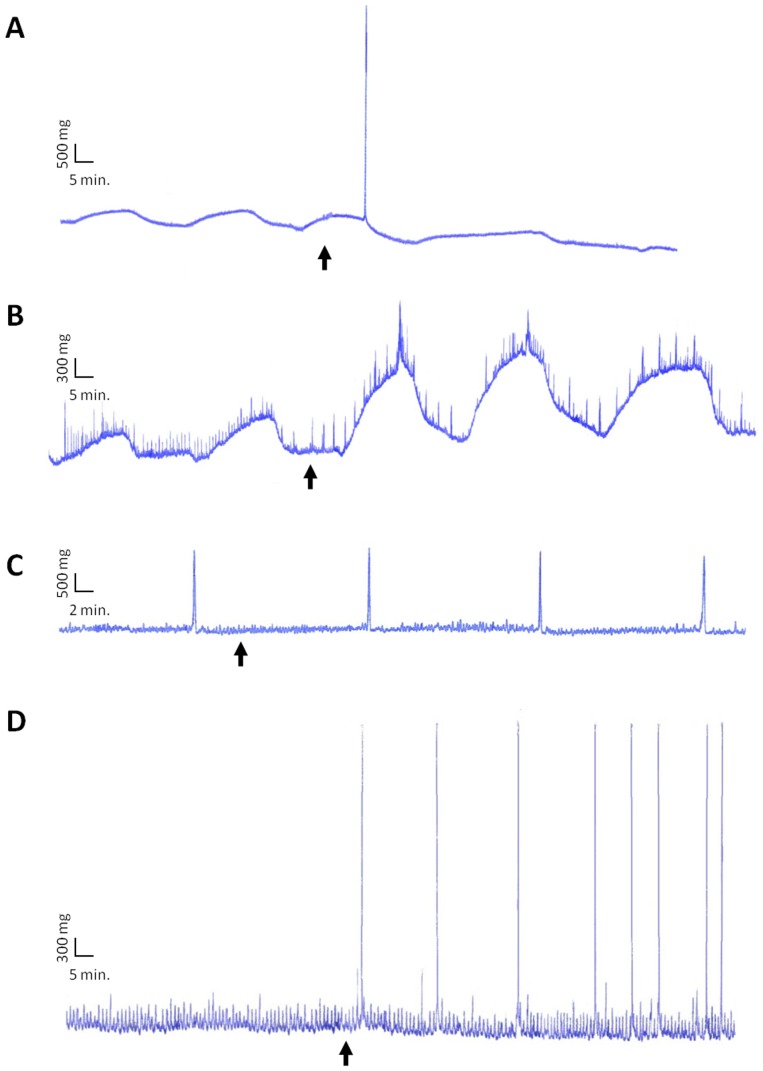
Bio-activity of synthetic β2 and α3 peptides. β2-induced contraction on (A) female gill and (B) penis from a threshold of 10^−8^ M. No activity on (C) rectum. α3-induced contraction on (D) penis from a threshold of 10^−9^ M.

The biological activity of the α3 and β2 peptides derived from SPα and SPβ respectively was checked using a myotropic bioassay. From as low as 10^−8^ M, the synthetic replicate of peptide β2 induced a strong contraction of the gills of the two sexes (figure 5A) and an increase in the basal tonus of the penis (figure 5B) whereas it had no effect when applied on the rectum of the two sexes (figure 5C), suggesting target specificity.

The synthetic replicate of peptide α3 was active on the penis only. It increased the amplitude of contractions from as low as 10^−9^ M (figure 5D).

## Discussion

Pheromones play an important role in the reproductive aggregation of marine invertebrate species. While they have been fully investigated in the marine gastropod *Aplysia* [16] [19] [20] [22] [26] [27], very little is known about the reproductive behavior of cephalopods. In this context, this study unravels one of the mechanisms that induce reproduction migrations of *S. officinalis* living in the English Channel.

*Aplysia* and *Sepia* aggregations occur every year in specific coastal areas. In *Aplysia*, the cocktail of polypeptides released by the albumen gland (a female accessory sex gland that secretes the egg capsule) and by the egg cordons induces *Aplysia* gathering and stimulates their mating [17] [22].

The occurrence of polypeptidic waterborne pheromones expressed and secreted by accessory sex glands was then highly suspected to account for the reproductive behavior of *S. officinalis*.

In order to identify large peptidic sex pheromones, we investigated the major transcripts of female *Sepia* accessory sex glands (which are physiologically similar to the albumen gland of *Aplysia*) expressing secreted peptides with a molecular mass over 2 kDa.

### General considerations about the EST strategy used in this work

Five hundred and seventy-six ESTs sequenced from a conventional cDNA library led to the identification of the main expression products. The ESTs were generated from accessory sex glands dissected during egg-laying, a specific narrow window of the life cycle, in order to focus on sex pheromones. These are the main expression products of the accessory sex glands during the short egg-capsule secretion period of marine mollusks such as *Aplysia* [19] [22]. In *S. officinalis*, EST analysis demonstrated a similar phenomenon in a cephalopod for the first time, with 268 clones encoding sexual pheromones out of 560 usable clones. Moreover, secreted products appeared as the main part of the expression products with 56 unique sequences (25.1%) out of 223 and three of them, SPα, SPα′ and SPβ representing 47.8% of the 560 sequenced clones. Quantitative analysis led to the conclusion that in egg-laying females most of the transcripts of accessory sex glands encoded secreted expression products.

In a similar study performed on male accessory sex glands of insects based on approximately 1,000 sequenced ESTs, gene ontology gave different results from ours concerning the ratio of molecular function categories (54% for *H. melpomene versus* 24% for *S. officinalis*), whereas the ratio of unique sequences encoding secreted peptides or proteins was similar (around 25%), as was the ratio of unknown unigenes (around one third of the total) [28].

### Sex pheromone precursors

Three major related transcripts encoding secreted peptides and expressed in the oviduct gland were identified. The similar SPα and SPα′ precursors, diverging by only four amino acids in the α3 and α3′ peptides, yielded seven putative expression products ranging from 1.3 kDa (peptide α5) to 7 kDa (peptides α3 and α3′). All peptides except α1 contained at least one cysteine and two of them, α3 and α3′, were C-terminally amidated like many bioactive peptides.

SPβ shared 56.7% similarity with SPα and SPα′ and yielded five putative expression products ranging from 1.1 kDa (peptide β1) to 8.3 kDa (peptide β3), with C-terminal amidation (peptide β2), disulfide bonds (peptides β3, β4, β5) or N-glycosylation (peptide β3).

For most of the expression products derived from SPα-α′ and SPβ, predicted post translational modifications such as C-terminal amidation, disulfide bonds or N-glycosylation could provide a strong protection against protease and peptidase activity and can be expected to confer the peptides a long life in marine environment. Thus, the processing of SPα-α′ and SPβ should lead to the release of a cocktail of waterborne pheromones.

In *Aplysia*, aggregation during the reproduction period is induced by the release of four waterborne pheromones processed from four distinct protein precursors [20] [21] [22]. In *Sepia*, the pheromone cocktail appeared to be more complex with at least nine putative pheromones resulting from dibasic cleavages (figure 2) and maybe more since peptides processed from internal cleavages of peptides α3 and α3′ were recovered by mass spectrometry. In accordance with these observations, a similar atypical processing was described for the Egg-laying hormone precursor in *Aplysia californica* [29].

Biological activity measured from the β2 and α3 peptides, two C-terminally amidated peptides, revealed few targets such as the penis and gills as compared to the expected functions associated to sex pheromones, i.e. mating stimulation and hyperventilation. Similar activities were also obtained from the egg-conditioning medium, demonstrating a release of pheromones by the egg mass (unpublished data). Behavioral tests will have to be performed with the cocktail of recombinant pheromones to investigate attractiveness on mature cuttlefish.

### Conclusion

In the present study, sex pheromones have been clearly identified by the use of a transcriptomic approach associated to structural and abundance criteria for the selection of ESTs. Although some of the predicted expression products were recovered by MALDI-TOF MS, the processing of precursors is not fully elucidated and has yet to be investigated.

However, we showed for the first time that sex pheromones from a cephalopod were expressed from three precursors in the oviduct gland during egg-laying. The sex pheromones of *S. officinalis* induce hyperventilation and stimulate mating. Compared with *Aplysia* sex pheromones, they have similar molecular masses and abundances of disulfide bonds, and similar biological activities. Nevertheless, protein precursors appear to be very different in the two *Sepia* and *Aplysia* genera. The processing of *Sepia* precursors yields a very complex set of predicted expression products that can be further cleaved or truncated (like α3 and α3′), leading to new sets of peptides or linked by multiple disulfide bonds, whereas *Aplysia* precursors (attractin, enticin, temptin, seductin) contain a signal peptide followed by a single expression product. We cannot rule out the occurrence of several precursor processing and the multifunctional involvement of expression products in cuttlefish.

## Methods

### Animal Handling and collection of cuttlefish glands

The two-year-old mature cuttlefish (*Sepia officinalis*) were trapped in the Bay of Seine in May and June, a location that is not privately-owned or protected in any way.

They were maintained in aerated natural seawater in 1,000-liter outflow tanks at the Marine Station of Luc-sur-Mer (University of Caen, France).

Female main nidamental glands, oviduct glands, gills, central nervous systems (NS), and male testis, secondary glands, penises, gills, and NSs were dissected from ethanol 3%-anesthetized mature cuttlefish [30], frozen in liquid nitrogen and stored at −80°C until RNA isolation or peptidic extraction. No specific permits were required for the described field studies and the common cuttlefish is not endangered or protected species.

### RNA extraction and construction of a cDNA library

Total RNA was isolated from oviduct glands or nidamental glands by the acidified phenol-guanidinium thiocyanate method [31]. Total RNAs from each gland were pooled at a 1∶1 ratio, and precipitated with lithium chloride (LiCl). Then, 4 µg of these total RNAs were used for ds-cDNA synthesis and amplification using the SMART approach [32].

The ds-cDNAs were directionally cloned into the pAL-17.3 plasmid vector (Evrogen, Moscow), and used to transform the XL1-Blue *E. coli* strain.

### Sequencing

Once bacterial library titration was completed (1.45×10^7^ bacteria per µl), a total of 576 clones were randomly picked and robotically arrayed in 96-well plates. Each clone was sequenced from the 5′ end using the T7 universal sequencing primer following plasmid DNA preparation. Sequencing was performed by MilleGen® Biotechnologies with BigDye V3.1 chemistries (Applied Biosystem).

### EST assembly

Raw single-pass sequence data were examined for possible sequencing errors. Open reading frames (ORFs) were manually inspected using the DNAsmac application (http://biofreesoftware.com/dnasmac). ESTs were trimmed from primer and vector sequences (using Vecscreen, http://www.ncbi.nlm.nih.gov/VecScreen/VecScreen.html), and poor quality sequences or sequences less than 100 bp in length were discarded. The inserted sequences were then clustered using the CAP3 assembly program (http://deepc2.psi.iastate.edu/aat/cap/cap.html) [33]. This software identifies ESTs representing the same transcripts after grouping all the ESTs into highly similar clusters. For the generated ESTs, if the similarity of a least 40 continuous bases was more than 90%, then the ESTs were theoretically regarded as fragments from the same mRNA or multi-copies of the same gene. They were assembled into a consensus sequence called a contig. The ESTs that could not be assembled with others were called singletons.

### Data analysis and annotation

An alignment of contig consensus sequences and singleton sequences resulting from the EST assembly process was performed against the NCBI non-redundant protein database using BLASTX (http://blast.ncbi.nlm.nih.gov/Blast.cgi) [34] [35] [36]. Only the homologous sequences that showed an E-value lower than 10^−5^ with more than 33 amino acids (or conserved cysteines) were considered for further analysis.

### Assignment of putative gene functions using Gene Ontology

Where possible, Gene Ontology (GO) classifications were assigned to each protein translation based on BLASTX (E-value<10^−5^) similarity to entries in a GO-annotated database (UNIPROT). GO annotations were summarized using ‘GO-Slim’ terms [37]. This process was automated using the Blast2GO (http://www.blast2go.org) [38] and InterproScan [39] operating systems. The top level consists of the following categories: biological process, cellular component, and molecular function. The second level divides these functional categories into finer sub-categories. Secretory signal sequence peptides were predicted with the SignalP software [25] [40].

### Expression products of SPα, SPα′ and SPβ

#### Recovery of material from tissues

For nanoLC-MALDI-TOF/TOF analysis, three main nidamental glands and three oviduct glands from mature animals were ground in liquid nitrogen, homogenized in 0.1% trifluoroacetic acid (TFA) at 4°C with a 1∶20 w/v ratio, and centrifuged 30 min at 35,000 g at 4°C. The supernatants were concentrated on Chromafix C18 cartridges (Macherey-Nagel).

#### NanoLC-MALDI-TOF/TOF MS

Dry pellets of acidic extracts were concentrated and desalted on OMIX-TIP C18 (10 µl, Varian), resuspended in 120 µl of 0.1% TFA in water. One hundred µl were pre-concentrated before being loaded on a C18 50 mm×75 µm capillary column and fractioned at a flow rate of 800 nl/min (nanoLC prominence, Shimadzu) with a 90-minute acetonitrile (ACN) gradient generated from 2 to 70% and leading to 360 15-second fractions mixed with the matrix and plated on the MALDI target (AccuSpot, Shimadzu). For each sample, two successive runs were performed to use two different matrixes: α-cyano-4-hydroxy cinnamic acid (CHCA) (5 mg of CHCA in 1 ml of 1∶1 ACN/0.1% TFA solution) for m/z ranging from 500 Da to approximately 3 kDa and sinapinic acid (SA) (5 mg of SA in 1 ml of 0.66∶1 ACN/0.1% TFA solution) for m/z over 3 kDa.

MALDI-TOF MS analyses were performed using a MALDI-TOF/TOF 5800 ABI SCIEX. Sample spots were submitted to multiple shots from the nitrogen laser (350 nm, 1000 Hz). The system was calibrated before analysis with a mixture of des-Arg-Bradykinin, Angiotensin I, Glu1-Fibrinopeptide B, ACTH (18–39), ACTH (7–38) and mass precision was better than 50 ppm in reflectron mode. In linear mode, the system was calibrated with a mixture of oxidized bovine Insulin β-chain, bovine Insulin, Aprotinin and Ubiquitin and mass precision was better than 200 ppm. The MS/MS spectra were acquired in reflector mode and screened using MASCOT (v2.3.02, Matrix Science LTD) against a translated EST library of female accessory sex glands for m/z under 3 kDa. For the m/z over 3 kDa, MALDI-TOF mass spectra were acquired in linear mode and the average masses of putative expression products were screened.

### Patterns of tissue-specific expression

We examined patterns of tissue-specific expression for unigenes of particular interest. Differences in expression were assayed via RT-PCR from 5 different tissues for each sex: ovary, main nidamental gland, oviduct gland, gill, central nervous system (NS) for females; and testis, secondary glands, penis, gill, NS for males.

Total RNA was isolated from each organ of mature female or mature male as described above.

RNA concentrations were determined using an ND-1000 spectrophotometer (Nanodrop Technologies) at 260 nm, using the conversion factor 1 OD = 40 µg/ml RNA. RNA integrity was verified on an Agilent bioanalyzer using RNA 6000 Nano kits (Agilent Technologies), according to manufacturer's instructions. A standard concentration of total RNA from each of these RNA extractions (1 µg) was treated with DNAse I RQ1 (Promega) and reverse transcribed into single stranded cDNA using random hexamer primers, MMLV-Reverse Transcriptase (Promega) and following the manufacturer's protocol. PCR primers were designed from the genes of interest (SPα, SPα′ and SPβ) and the reference gene (Actin), within the predicted ORF : SPα/α′-s (5′ CACGACTCTGAGGTCGCTCGTAATC 3′), SPα/α′-a (5′CACAGCAGGGAACTTCTTTGT3′), SPβ-s (5′TTCTGGCCCTTATTTTCGTG3′), SPβ-a (5′ CGGCAATTCAAACCCATTAC), Act-s (5′TCCATCATGAAGTGCGATGT3′), Act-a (5′TGGACCGGACTCGTCATATT3′) as sense and antisense primers, respectively.

SPα/α′ primers were designed inside the region coding for α3-3′ and SPβ primers inside the region coding for β2.

One µl of this cDNA was used as a template in a 50-µl multiplex PCR (MyCycler, Bio Rad), targeted to the with the following cycling parameters: initial denaturation at 95°C (10 min), 30 cycles at 95°C (45 sec), then 56°C (45 sec), and then 72°C (1 min), and a final extension at 72°C (10 min). For each set of primers an equal amount of PCR amplicon (6 µl) from each of the ten templates was electrophoresed at 100 V for 30 min in 1.8% agarose gel, stained with ethidium bromide and visualized under UV light (alphaimager EP, alpha Innotech). The target-specific amplified products had expected sizes of 656 and 785 bp for SPβ or SPα/α′, respectively, and 261 bp for Actin.

### Synthesis of peptide β2 (2.3 kDa)

The β2 peptide (FTYPYVQVKIPGPGATYVIWamide) was synthesized on a 0.1 mmol scale by employing the Fmoc (*N*-[9-fluorenyl]methoxycarbonyl)/*t*Bu (*tert*-Butyl) SPPS strategy using a microwave peptide synthesizer with UV monitoring (Liberty, CEM), by coupling Fmoc-α-amino acids on Rink amide resin (0.56 mmol/g). All microwave-assisted coupling reactions were performed (300 s, 25 W, 70°C) with 4 eq of Fmoc-amino acids, 4 eq of 2-(1H-Benzotriazole-1-yl)-1,1,3,3-tetramethylaminium tetrafluoroborate (TBTU) and 10 eq of diisopropylethylamine (DiPEA) in DMF. A coupling time of 5 min was used. *N*α-Fmoc deprotection was performed with 20% piperidine in dimethylformamide (DMF). Side chain deprotection and cleavage of the peptide from the solid support were performed by treatment with 95% trifluoroacetic acid (TFA)/2.5% triisopropylsilane (TIS)/2.5% water for 3 h at room temperature [41]. The resin was filtrated and the crude product was precipitated in tert-butylmethylether and filtrated. The crude peptide was purified by reversed-phase HPLC (RP-HPLC) using a Waters semi-preparative HPLC system with an X Terra 10 µm column (300 mm×19 mm). The elution was achieved with a linear gradient of aqueous 0.08% TFA (A) and 0.1% TFA in acetonitrile (B) at a flow rate of 10 ml/min (5–60% B over 30 min) with photodiode array detection at 210–440 nm. The purity of the peptide (>95%) was controlled by analytical RP-HPLC on the same instrument with an X Terra 5 µm column (250 mm×4.6 mm) using a linear gradient of 0.08% TFA in water and acetonitrile containing 0.1% TFA at a flow rate of 1 ml/min (5–60% B over 20 min). Finally, integrity of the peptide was assessed by MALDI-TOF/TOF analysis.

### Synthesis and oxidization of the α3 peptide (6.9 kDa)

The α3 peptide (PTWANLKGGSQLGGLIYGTQYKLYPAHVNGFGLFITTHLKPPHNHDVPCAVCQVTK PATVLMIP-NH_2_) was synthesized (0.1 mmol scale) on a Rink amide MBHA resin (VWR, Fontenay-sous-Bois, France) using a Liberty Microwave Peptide Synthesizer with UV monitoring (CEM, Orsay, France) and following the manufacturer's standard procedure. All microwave-assisted coupling reactions were performed (300 s, 25 W, 70°C except for Cys and His : 120 s, 0 W, 50°C then 240 s, 25 W, 50°C) with 5 eq of Fmoc-amino acids (Senn Chemicals, Dielsdorf, Switzerland), 5 eq HBTU (VWR) and 10 eq DIEA in DMF (Fisher Scientific, Illkirch, France). Double coupling reactions were used for Thr^2^, Trp^3^, Leu^15^, His^27^, Val^28^, Leu^33^, Ile^35^, His^43^, Asn^44^, Val^47^, Cys^49^, Ala^50^, Val^51^, Cys^52^, Gln^53^, Val^54^, Thr^59^ and Val^60^. Peptide cleavage from the resin and deprotection of the amino acid side-chains were carried out with a TFA/thioanisole/phenol/H_2_O/EDT mixture (82.5∶5∶5∶5∶2.5, v/v/v/v/v, Sigma-Aldrich, Saint-Quentin-Fallavier, France, each) for 3 h under agitation at room temperature. The resin was filtrated and the crude product was precipitated in tert-butylmethylether (Sigma-Aldrich) and collected by centrifugation (15 min, 4,500 rpm) as previously described [42] [43] [44]. The crude peptide (reduced form) was purified by reversed-phase HPLC (RP-HPLC) on a Vydac 218TP1022 C_18_ column (2.2×25 cm; Grace Discovery Sciences Alltech, Templemars, France) using a linear gradient (20–50% over 45 min) of ACN/TFA (99.9∶0.1, v/v) at a flow rate of 10 ml/min. Oxidization of the peptide was carried out with DMSO. Briefly, the peptide was solubilized (0.5 mM) in acetic acid (5%, pH = 6) and shaken for 22 hrs at room temperature after addition of DMSO (20% of the final volume). The solution was diluted in H_2_O and freeze-dried. The α3 peptide was purified by semi-preparative RP-HPLC using the same chromatographic conditions as described above. Analytical RP-HPLC analysis, performed on a Vydac 218TP54 C_18_ column (0.46 cm×25 cm, Grace Discovery Sciences Alltech), revealed that the purity of the oxidized peptide was 83.6% (16.4% of reduced peptide). The authenticity of the α3 peptide was controled by MALDI-TOF/TOF on a Voyager-DE PRO (AB SCIEX, Les Ulis, France) in the linear mode with sinapinic acid as a matrix.

### Biological assay

A preliminary screening of contractile organs was performed using a myotropic bioassay. Gills, rectum and penis were dissected from mature cuttlefish anesthetized with ethanol 3%. Each organ was suspended to a displacement transducer (Phymep, Bionic Instruments) connected to a printer. The muscle chamber was perfused at a flow rate of 0.5 ml/min with synthetic seawater (Instant Ocean) containing 1 mM glucose and maintained at 15°C. The samples were re-suspended in 100 µl of perfusion medium and injected using a three-way valve in order to avoid mechanical and thermal stress. The flow of samples into the muscle chamber was traced by addition of phenol red. No specific permits were required for the described field studies.
